# The Text-mining based PubChem Bioassay neighboring analysis

**DOI:** 10.1186/1471-2105-11-549

**Published:** 2010-11-08

**Authors:** Lianyi Han, Tugba O Suzek, Yanli Wang, Steve H Bryant

**Affiliations:** 1 National Center for Biotechnology Information, U.S. National Library of Medicine, 8600 Rockville Pike, Bethesda, MD 20894, USA

## Abstract

**Background:**

In recent years, the number of High Throughput Screening (HTS) assays deposited in PubChem has grown quickly. As a result, the volume of both the structured information (i.e. molecular structure, bioactivities) and the unstructured information (such as descriptions of bioassay experiments), has been increasing exponentially. As a result, it has become even more demanding and challenging to efficiently assemble the bioactivity data by mining the huge amount of information to identify and interpret the relationships among the diversified bioassay experiments. In this work, we propose a text-mining based approach for bioassay neighboring analysis from the unstructured text descriptions contained in the PubChem BioAssay database.

**Results:**

The neighboring analysis is achieved by evaluating the cosine scores of each bioassay pair and fraction of overlaps among the human-curated neighbors. Our results from the cosine score distribution analysis and assay neighbor clustering analysis on all PubChem bioassays suggest that strong correlations among the bioassays can be identified from their conceptual relevance. A comparison with other existing assay neighboring methods suggests that the text-mining based bioassay neighboring approach provides meaningful linkages among the PubChem bioassays, and complements the existing methods by identifying additional relationships among the bioassay entries.

**Conclusions:**

The text-mining based bioassay neighboring analysis is efficient for correlating bioassays and studying different aspects of a biological process, which are otherwise difficult to achieve by existing neighboring procedures due to the lack of specific annotations and structured information. It is suggested that the text-mining based bioassay neighboring analysis can be used as a standalone or as a complementary tool for the PubChem bioassay neighboring process to enable efficient integration of assay results and generate hypotheses for the discovery of bioactivities of the tested reagents.

## Background

The number of High Throughput Screening (HTS) assays deposited in PubChem [1] has grown quickly in recent years. As of April 18th 2010, PubChem has over 2300 bioassay records that are publicly available, including primary HTS assays and confirmatory assays associated with hundreds of targets or cell lines. While a number of tools have been developed to utilize the chemical structure information and the bioactivity outcomes[2], the large volume of textual descriptions for assay protocols has made it a very challenging task to analyze and interpret such unstructured information toward the new and structured information.

One systematic knowledge driven study of HTS assay data is to understand how they are inter-related. The PubChem database currently provides four methods of identifying bioassay relationships, which are based on 1) target information, 2) commonly tested active compounds, 3) commonly participated biological pathways, and 4) depositor annotations respectively [1]. The utilization of the target sequence similarity analysis enables one to find related bioassays that share biologically related targets. Compound activity based assay neighboring procedure allows one to cluster assays based on activity profile similarity measured by commonly tested compounds. Pathway based approach groups together the bioassays if their targets involved in a common biological pathway. Depositor specified assay relationships are provided by the contributors of the bioassay entries when the assays are submitted to PubChem. There are various limitations of the existing methods, as they depend on the unambiguous identification of either the sequence information or the molecular pathways of the assay targets, or otherwise depend on the provision of comprehensive annotations by depositors, which is lacking in many bioassay records. As a result, a noticeable amount of relationships among the bioassays has not been captured by existing approaches. On the other hand, there is a great amount of meaningful information stored as unstructured free text in the bioassay descriptions which is not being utilized by the existing neighboring approaches (such as objectives of the assays and detailed information about the experimental protocols). With the rapid growth of the PubChem BioAssay database, the ability to pool such unstructured information from related biological tests together has become increasingly important for getting insights into biological processes. Therefore, identifying assay relationships by utilizing the textual bioassay descriptions is crucial for improving the usability of PubChem.

Text-mining based knowledge discovery has proven to be a complex task due to the ambiguity, complexity and domain specific aspects of the real world [3]. Text-mining based approach encounters various specific problems and great challenges, such as the complexity of biomedical nomenclature. Notable attention has been paid in text-mining biomedical literature, databases and documentations. Efforts have been made in several directions to detect, distinguish, extract and interpret relevant information [3,4].

With the advances in text-mining techniques, one can identify and extract the desired content and make the document structurally organized, indexed and computationally accessible. For example, using the detection of the protein name and gene names [4-10], or the extraction of chemical names [11,12], one can build and maintain an information extraction (IE) system for characterizing the biological events like protein-protein interactions[13-18]. Statistical models can also be applied to help generate new hypotheses, and mine the data of interest in cases where the knowledge domain is not pre-defined or is defined too loosely.

The text-mining approach utilizing the frequency of the selected terms is straightforward, and has proved effective in the analysis of biomedical literature. For example, several independent research groups have succeeded in the mining of gene expressions based on free text [19-23]. The use of co-occurring frequencies together with the machine learning approach has been successfully applied in tackling many bioinformatic problems, such as those for predicting protein sub-cellular localizations [24-27], probing protein-protein interactions [28], and providing protein function annotations [29-31].

In this work, we propose a new PubChem bioassay neighboring analysis that uses a text-mining based approach. Given our primary interest to find bioassay relationships, this approach utilizes the free bioassay descriptions combined with the Bag-of-words (BOW) approach for the numerical understanding of these text descriptions. Bioassay descriptions can be represented by a collection of lexical features or BOW. Therefore, they can be conceptually compared by the collections of word terms contained, which can be used to both identify the relationships among the bioassays and to measure their relevance. In this article, we show that this text-mining based neighboring analysis provides encouraging results by focusing on the overall conceptual content of the assay descriptions. It has the potential to group biologically relevant bioassays in a broader context, and complements with the existing assay neighboring methods.

## Methods

### Corpora Analysis

The Corpora analyzed in this work is the depositor provided assay descriptions retrieved from the PubChem BioAssay database. The unified description usually contains important information (such as the biological background of an assay experiment, the biological system involved, the experimental procedures, and its relevance to disease and therapeutic treatment). Since the content is very flexible and there is no controlled dictionary that can be used for the conceptual understanding of the assay descriptions, the BOW approach is implemented to mine the collection of words that are meaningful in describing the bioassays. Extracting the unique terms or tokens of interest can be obtained by doing the following: 1) preprocessing the assay descriptions, 2) filtering out punctuation and a pre-collected set of stop words, and 3) removing parentheses and extra spaces. Stop words, such as 'is' and 'this', are usually less meaningful to the concept of the document, therefore are excluded during the tokenizing step and every term token obtained from assay descriptions is stemmed to their unified form for the purpose of accurate comparisons by using the Porter stemming algorithm[32]. For instance, "cell" and "cells" will have no difference in terms of their concept. Hyphenated words are not split for preserving chemical names and formulas as single entities.

### Feature Generation

The keyword terms of interest can vary in each of the individual assay descriptions. One computational approach to measure the difference is to compute their "occurrence frequency" and "document frequency".

The "occurrence frequency" of a certain term usually means "the number of occurrences of the term in one document". And the "document frequency" usually means "the total number of occurrences of a given term in a set of documents".

The document frequency was represented by the inverse document frequency (IDF)[33],

$$IDF_{i,j}=\log_{2}\frac{M_{i}}{m_{i,j}}$$

where i is the index of the given term, j is the index of the document. *M*_*i *_is the total number of the occurrence of term i, and *m*_*i,j *_is the number of occurrence of term i in the j-th document.

The document analysis process is designed to iterate each assay description and create a vector of the term occurrences for each unique word. Thus a document vector *Vd *is defined as:

$$Vd_{i}=\left(v_{1,i},v_{2,i},...,v_{k,i}\right)$$

where *k *reflects the number of unique words of interest that are collected after the pre-processing, *v_j,i _*is defined by the combination of the occurrence frequency and document frequency [19,34,35]:

$$v_{j,i}=\frac{1}{2}(1+norm(m_{i,j}))IDF_{j,i}$$

where *norm*(*m*_*i,j*_) is the normalized occurrence frequency in the document j.

Hence, *v*_*j,i *_could be used to reflect the importance of a word term in two ways. First, it will have a higher value if the frequency of a word term in one assay description is higher and secondly it will have a lower value when the word term is co-occurring in more bioassays.

### Similarity Measurements

The measurement of similarity between a pair of assay descriptions was calculated by comparing the two document vectors. There are a number of ways to compute the similarities among vectors, such as Euclidean Distance(ED) and Cosine Angle Distance(CAD), which is the cosine of the angle formed by the two document vectors. Both ED and CAD are widely used and work similarly for neighboring analysis of high dimension vectors[36]. In this work, we chose the CAD for its naturally normalized values. The cosine score of two assays will be zero if they have nothing in common in their descriptions, whereas the score will be 1 if the two assays have identical descriptions.

$$Similarity_{i,j}=cosine(V_{i},V_{j})=\frac{V_{i}•V_{j}}{\left\|V_{i}\right\|\times \left\|V_{j}\right\|}$$

where *V *is a document vector.

### Clustering Analysis

We performed an unsupervised single linkage clustering analysis for the bioassays within PubChem, which was conducted by simple binning or neighboring the document vectors based on the pairwise similarity. We adopted an unsupervised clustering procedure here as it does not rely on predefined training examples while clustering the data objects. This clustering approach is different from pattern recognition or the areas of statistics known as discriminate analysis and decision analysis, as it was not aimed to maximize a utility function but rather to find similarities among the data objects.

Evaluation of such unsupervised clusters extracted from medical text is shown to be problematic and external help (such as expert opinion) is often required [37]. In the following section, we are going to describe an evaluation scheme based on a human-curated subset of bioassay neighbors used as "expert opinion".

### Results Evaluation

To evaluate the results of the text-mining based neighboring method, we first generate the distribution of the cosine scores over all pairs of bioassay descriptions to determine the threshold for suggesting meaningful relationships, and evaluate its sensitivity to the textual content of the bioassay records.

For the second type of evaluation, we computed the overlap of the bioassay neighbors result from the text-clustering method against the human-curated neighbor set (as provided by the depositor) in the PubChem BioAssay database. Depositor-specified bioassay neighbors were determined by assay data providers to address various aspects of assay relationships (such as to link primary and confirmatory assays of the same project, designate counter screenings for alternative targets, or measure other properties of the primary hit compounds). PubChem allows a depositor to cite assays from other depositors if that helps to illustrate their data or support their conclusion, though most of the depositor-provided assay neighbors were from the same depositor.

## Results and Discussion

A total number of 2,322 PubChem bioassays were used in this study. The contents of the descriptions are unstructured texts that usually provide background information about the goal of the screening (such as the biological system used in the research, the significance of finding molecular modulators and their relevance to disease treatment, and experimental protocols). Assay descriptions were extracted from the PubChem BioAssay database, for which the BOW analysis was conducted.

### Cosine score distribution and clustering analysis

**Figure 1:**
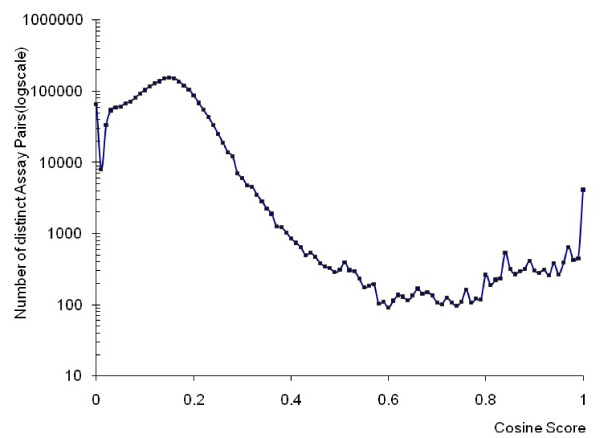
**Cosine score distribution over bioassays**.

The distribution of the cosine scores over all pairs of bioassay descriptions is provided in Figure 1. Two major separated areas are observed where most of the cosine scores of the studied assay pairs fall. About 95% assay pairs fall in an area with a cosine value between 0 and 0.5. Another relative small area is located in the region with the cosine value between 0.8 to 1. As it can be seen from the distribution plot (Figure 1), a border line between these two regions can be drawn clearly between cosine values of 0.4 and 0.8. Since a lower cosine value of an assay pair resulting from the text-mining analysis suggests a weak relevance among the two assays, a cosine score in the range of 0~0.4 probably suggests a random relationship or weak relevance. On the other hand, a cosine value ≥ 0.8 likely gives strong indication of high relevance among the assays compared. Nearly 0.5% of all possible assay pairs fall in this region, which suggests interesting relationships among these bioassays. The region with a cosine score in the range of 0.4 ~ 0.8 represents a small fraction (less than 1%) of assay pairs.

To examine the assay relevance measured by the cosine score, a manual verification and spot checking process was conducted by curating the details of the assay descriptions. The overall verification of the assay pairs reveals that the majorities of the identified assay neighbors with higher cosine score are closely related, and suggests that the cosine score serves as a strong indicator of conceptual relevance among bioassays. In particular, our result shows that the assay pairs with cosine score 0.90 or higher are directly related. For example, Bioassay AID 777 and AID 778 are identified as highly related (cosine score 0.94) in spite of the fact that their studies were on cytochrome P450 enzymes with different metabolic functions. The goal and protocol of these two assays were quite similar, which was to test the ability of the compounds to inhibit members of the P450 enzyme family for the conversion of the substrate luciferin-H EGE to luciferin EGE. By recognizing such related assays, one would be able to combine the results from the counter screenings to evaluate the inhibition specificity of the compounds towards different members of cytochrome P450 families. Assay pairs with a cosine score of 0.8~0.90 can also be highly related.

When looking into the twilight zone with cosine score range of 0.5 ~0.8, it was noticed that identification of assay pairs with a cosine score greater than 0.6 can sometimes also be of interest to reveal their biological relevance. For the set of cellular toxicity HTS assays that the NIH Chemical Genomics Center (NCGC) developed against a number of cell lines, the text-mining based method was able to group a great portion of these assays together. For example, AID 658, AID 659, AID 661 and AID 657 were clustered together as they were all designed for measuring compound cellular toxicity in human cell lines. Meanwhile AID 433, 543,540, which were designed to determine *in vitro *cytotoxicity, were also clustered together. The potential to cluster such assays with toxicity measurement could be useful to construct an assay panel to systematically analyze the toxicity profiles of the compounds across multiple cell lines or organisms.

### Comparison of the text-mining based neighbors against the human-curated set

The human-curated bioassay neighbor set refers to the related bioassays annotated by depositors. Depositor-specified annotations were subjected to the examination of the PubChem curators during the bioassay deposition process. Depositor-specified related bioassays address various aspects of assay relationships, such as linking primary, confirmatory and counter screenings of the same assay project. Although the perspective of the depositors may vary, such annotations on bioassay relationships provide a benchmark for evaluating the recognition of bioassay relationships by the text-mining algorithm. The selection of cosine score threshold is critical for identifying significant relationships among the compared assays. There is a trade-off between the precision and the recall for optimizing the threshold. Therefore, it is essential to compare the performance of identifying related assay pairs at a series of cosine score thresholds. The result of this analysis using the depositor provided bioassay relationship as a benchmark is summarized in Table 1, where "precision" was defined as the ratio of true predictions over the complete predictions, whereas "recall" was defined as the ratio of true predictions over the depositor defined neighboring pairs. "True prediction" was defined as the overlap between the depositor defined neighboring pairs and those predicted by the text-mining method.

**Table 1:** A summary of precision and recall under various cosine score threshold by comparing the text-mining based neighbors and depositor-specified neighbors.

Cosine similarity cut-off	Precision	Recall
**0.1**	0.003	0.99
**0.2**	0.013	0.98
**0.3**	0.082	0.92
**0.4**	0.150	0.86
**0.5**	0.169	0.79
**0.6**	0.173	0.73
**0.7**	0.163	0.63
**0.8**	0.155	0.52
**0.9**	0.146	0.33

This analysis suggests that cosine score of 0.4 can serve as a reasonable cut-off to balance the precision and recall, which agrees with the earlier analysis about the cosine score distribution. It is noted that precision at a threshold of cosine score of 0.9 is low. This is due to the limitations of the human-curated assay neighboring set where the coverage is low or incomplete. This is the case especially for the assays contributed by the NIH Molecular Libraries Program (MLP), where reports for a specific assay project are often split into many bioassay records, mostly because an assay project including follow-up experiments may take a few months to a couple of years to complete. Data produced at each experimental progress are required to be deposited in a timely manner into the central PubChem repository. Sometimes depositors tend to deposit test results from different compound libraries or from counter screenings under separate records. Thus, tracking the deposited records and providing a comprehensive linkage annotation on the overall assay relationship are burdensome for depositors, which explains one reason for the lack of a complete bioassay linkage annotation from depositor in PubChem.

A significant amount of assay relationships can be confirmed by examining the assay pairs identified by the text-mining approach through spot checking. Although these assay pairs were not specified as related at the deposition time, about 99% of the assay pairs identified at this threshold were deposited by the same assay providers. It suggests that the text-mining based method complements human annotations significantly when only a limited bioassay relationship is provided by depositors.

In many cases, our analysis also suggests that neighboring relationships from text-mining based bioassays correlate well with intrinsic relationships among bioassays. Moreover, this approach is especially efficient under conditions where other assay-clustering methods encounter limitations to apply. Bioassays AID 454, 455, 456, and 457 are related for screening compounds for enhancing/attenuating TNFa induced VCAM-1 cell surface expression with AID 457 (imaging assay) and 455 (plate reader assay) reporting compounds with augmentation effect, and AID 456 (imaging assay) and 454 (plate reader assay) reporting compounds with inhibition effect. Identifying the significant relationships among these assays would allow one to collect effective chemical reagents for the studied biological process. Unfortunately, such a relationship was not annotated by the assay depositors, and none of the other three automated assay neighboring approaches could detect this relationship due to the lack of target specification or common hits. However, with the aid of the text-mining based approach, the biologically important relevance among this group of assays were successfully identified. As another example, primary assays (AID 738, 739, 636,637) searching modulators of post-Golgi transport were first clustered together at a cosine score cut-off of 0.90, then further connected with the related dose response assays(AID 788, 789, 790) at the cosine score cut-off 0.88. This hierarchical clustering result reflects the biological relationships among the assays at three levels: the purpose of the assays, the experiment and project stage. None of these bioassays have protein target information and they have very limited active compounds in common. Thus it is very difficult for the existing automated neighboring methods to discover their relevance.

The text-mining approach compares each bioassay in PubChem against all of the rest bioassays irrespective of the data source. While this method is mostly efficient for detecting the relationship among assays from the same depositor, it was observed that nearly 1% of the related assays pairs identified are from different depositors. One such example is the AID 465 and 819 pair. These two assays came from two data sources but were recognized as related assays by the text-mining based method. Both assays were set up to identify chemicals modulating NFkB activities. Neither of them have targets defined, thus again making the existing target based neighboring method not applicable to them.

### Comparison among assay-neighboring analysis approaches

There are different interests and perspectives when the enormous collection of HTS data is interpreted. Currently, there are four approaches in PubChem for neighboring bioassays, with each providing different insights into the bioassay relationship. These approaches include three automated approaches by using common biological pathway, finding sequence homology among protein targets, calculating chemical structure identity among hit compounds, and the one using the annotations from bioassay depositors.

For evaluating the new text-mining based approach, comparisons of the four automated neighboring procedures were performed and summarized below using the human annotations as a baseline.

### Target similarity based bioassay neighboring

Target similarity based bioassay neighboring analysis enables one to identify assays tested against biologically related molecular targets, facilitating the construction of an assay panel for compound selectivity and specificity study. The relevance of bioassays is evaluated by the sequence similarities of their protein targets. This approach is both simple and effective in clustering bioassays. It enables the straightforward retrieval and comparison of the sequences of the assay targets. The BLASTP[38] algorithm was employed to identify the homology between bioassay targets. On the other hand, the target similarity based neighboring analysis can only be applicable to the bioassays for which protein targets are explicitly defined. As about 40% of the bioassays in the PubChem do not contain a protein target, the target-based neighboring approach will not work for these bioassays.

### Activity overlap based bioassays neighboring

An individual HTS assay for small molecules usually measures certain bioactivity properties as well as describes the bioactivity outcome for the tested compounds in a specific biological system. In order to decide whether a follow-up study is worthwhile, a compound may be tested in multiple HTS screenings assays that share common active compounds together would facilitate a comparison across multiple assays. In addition, a common group of compounds that perform similarly among different assays can be a very interesting indicator of the underlying relationship between the biological system used or the biological process monitored in the assays. Therefore the assay relationship identified by checking activity overlap or common hits could be of interest for generating new hypotheses. However, this approach is sensitive to the selection of the compound libraries tested in the assays, and may not be applicable for every assay. Since each assay may test a specific compound library, the overlap among the compound libraries is apparently the first determinant factor for this approach when neighboring assays. In addition, this method is also prone to experimental noise from HTS screenings.

### Common biosystem based bioassay neighboring

In the biosystem based assay neighboring method, common biological pathways of the respective proteins or gene targets are examined. The bioassays are considered as related if their protein or gene targets participate in the same biological pathways by using the National Center for Biotechnology Information BioSystems database [39]. This type of relationship allows one to aggregate assay results and to identify the compounds affecting a common pathway. Similarly to the target homology based assay neighboring method, this approach relies on unambiguous annotations for the assay targets or the molecular pathways studied.

### Text-mining based bioassay neighboring

Unlike the other bioassay neighboring methods discussed above, the text-mining based approach does not rely on the availability of specific annotations, but utilizes the free text descriptions. Since there is no specific domain knowledge defined prior to the text-mining, the relevance of bioassays depends on the concept of descriptions. Here the underlying concept of descriptions could be the accumulation of multiple meaningful terms (such as the description about a biological process, name of the protein or gene involved, HTS screening protocol, activity type and assay readout, or methods for activity measurement).

### Analysis of result comparison

One of the advantages of the text-mining based neighboring analysis is to discover the relevance that other automated approaches cannot. To provide further evaluation of the text-mining based approach, the assay neighbors identified by this method were compared to those annotated by the bioassay depositors and those suggested by the other three automated methods. Precision and recall values for each automated method were computed using the depositor provided neighbors, which is also known as human-curated assay neighbors, as the benchmark. This dataset is contributed by independent bioassay submitters, which represents expert opinions upon the pairwise bioassay relationships. As this benchmark set does not depend on any particular data elements as required by the automated methods, it provides a way to examine to what extent the automated neighboring methods are in agreement with the human curated dataset and with each other. Annotations of the depositor provided related bioassays are stored in each bioassay record (query assay) in the PubChem database if applicable. In each such annotation, one assay can be denoted to be related to one or more assays (neighbor assays) through the cross-reference data field, resulting in one or more assay pairs for each of such annotation. To construct the benchmark dataset, related assay pairs were extracted from a total of 1747 bioassay records for which the depositor provided annotations are available. These assay pairs were further grouped into 1306 clusters using the unsupervised single linkage clustering procedure. The final list of assay pairs was derived by considering all possible pairwise combinations of the assays deemed related by each clustering method, and the total number of assay pairs were derived accordingly. As a result, the benchmark dataset contained 8802 bioassay pairs, with 41% of the assay pairs containing no target information. The median cluster size was 4 and 216 clusters containing a single assay pair. The F1 score, the harmonic mean of the precision and recall values, was provided for clearer comparison of the overall performance of the methods compared to human curated datasets. In this analysis, the cosine similarity threshold for the text-based method was 0.4. The results are summarized in Table 2. Among all four automated methods, the text-mining based approach apparently has the best recall and precision compared to the depositor-specified neighbors.

**Table 2:** Comparison of the four automated bioassay neighboring methods by using the depositor-defined (human-curated) assay neighbors as a benchmark.

Neighboring method	Recall	Precision	F1 score
	Number of common pairs/Number of depositor-specified pairs	Number of common pairs/Number of assay pairs by the method	Harmonic mean(recall, precision)
**1. Activity based**	41% [3694/8802]	0.5% [4100/671799]	0.99%

**2. Target similarity based**	46% [4100/8802]	0.6% [4100/66497]	1.18%

**3. Biosystems based**	34% [3008/8802]	0.2% [3008/160356]	0.39%

4. **Text-mining based**	**86% [7530/8802]**	**15.0% [7530/50148]**	**25.50%**

It can be observed from Table 2 that the three existing automated methods perform similarly well. The recall values for those three methods which are in the range between 34% and 46% are reasonable and understandable given the intrinsic limitations within these methods as discussed previously and intrinsic nature of the dataset. About 41% of the neighbor pairs in the benchmark dataset involve cell or organism based assays and contain no target. Thus, target and biosystems based methods are not able to detect this considerable portion of the bioassay relationships. On the other hand, the text-based method is not bound to any particular structured data, thus is able to recognize the relationships even among the cell or organism based bioassays. The significantly higher recall value (86%) indicates that this approach complements the existing methods remarkably.

The low precisions, which led to low F1 value, for all four methods were expected. As discussed earlier for the results shown in Table 1, this was largely due to the low coverage of the depositor-provided relationships. While the human curated dataset is highly reliable for deriving bioassay relationships, it has been observed that its coverage is rather limited and a great portion of true and meaningful relationships are not fully captured, which motivated the development of alternative approaches for detecting bioassay relationships including the text-mining based approach in this work. As discussed previously, the limited coverage is partly due to the fact that bioassay submissions from the same or related projects can be done over an extensive period of time; it is troublesome for depositor to track the submissions. As a result, assay depositors sometimes neglect to provide comprehensive annotations for the bioassay relationship even for assays from the same project. Secondly, individual bioassay depositors from different laboratories may work on assays against the same or biologically related targets. Since assays from different data sources are submitted to PubChem independently, the relationship among such assays is typically not recognized by the depositors. This explains why the automated methods, particularly the target and biosystems based methods, are detecting many folds of additional and meaningful bioassay relationships which literally led to low precisions. We considered the related assays from the target and biosystems based methods containing true biological relationships as these were resulted from conservative analysis based on biological sequence of targets involved in the assays. *i.e *there were true predictions but not annotated by the depositors. When looking into the overlap of the predictions among different methods, 62.5% of the assay pairs from the text-mining approach were further confirmed by at least one of the other automated procedures, indicating that these bioassays can be related one way or another. Furthermore, the majority of the novel pairs detected by the text-based method involve assays contained no target specifications, which again demonstrates that the text-based approach may play a critical role in detecting bioassay relationships that are otherwise impossible for the existing methods to recognize.

## Conclusion

In this study, we proposed a text mining based approach for neighboring PubChem bioassays from their unstructured text descriptions. The conceptual understanding of the bioassays is implemented by the BOW method, which extracts all of the meaningful word terms and their descriptors and represents the contextual importance of the words. The distribution of the similarity, measured by the cosine scores on all PubChem bioassay pairs, is obtained for deriving a meaningful and practical threshold and evaluating the precision and recall of the proposed neighboring analysis approach. This analysis indicates that a cosine score of 0.8 and above would suggest higher relevance for the compared bioassays which is confirmed by manual verification. The analysis on the precision and recall suggests that a threshold at cosine score 0.4 or higher may be used to identify a significant assay relationship. A statistical analysis using this threshold shows an overlap of 86% between the related bioassays detected by the text-mining approach and those specified by depositors. Moreover, this neighboring analysis identifies a significant fraction of additional assay relationships that were missed by depositors. Furthermore, results of the text based approach were compared to those derived by three other automated neighboring procedures based on target similarity, bioactivity overlap and common pathway. This comparison suggested that the text-mining neighboring analysis provides a meaningful approach based on the perspective of descriptive content in the bioassay records. The text-mining based bioassay neighboring analyses has proven to be advantageous and efficient particularly for correlating bioassays for studying different aspects of a biological process, which otherwise are difficult to achieve by the existing neighboring procedures due to the lack of specific data elements required by those neighboring methods. It also allows one to prioritize the assay relationship to facilitate the information retrieval. Overall, the text-mining based bioassay neighboring analysis can be used as a standalone or as a complementary tool to the PubChem bioassay neighboring process to enable efficient integration of the assay results and improve the utility of PubChem.

## Availability and Requirements

The PubChem database is available at http://pubchem.ncbi.nlm.nih.gov/. A latest web browser with JavaScript enabled is required to use it. The PubChem Bioassay data is also available at ftp://ftp.ncbi.nlm.nih.gov/pubchem/Bioassay/ through FTP. PubChem bioassay neighboring analysis data can be downloaded at ftp://ftp.ncbi.nlm.nih.gov/pubchem/Bioassay/AssayNeighbors/.

## Authors' contributions

LH and TS carried out the bioassays neighboring analysis, participated in its design and performed the statistical analysis. YW and SB conceived of the study, and participated in the design, project coordination and data analysis. All authors read and approved the final manuscript.
